# Statistical independence in nonlinear model-based inversion for quantitative photoacoustic tomography

**DOI:** 10.1364/BOE.8.005297

**Published:** 2017-10-27

**Authors:** Lu An, Teedah Saratoon, Martina Fonseca, Robert Ellwood, Ben Cox

**Affiliations:** Department of Medical Physics and Biomedical Engineering, University College London, Gower Street, WC1E 6BT, UK

**Keywords:** (170.5120) Photoacoustic imaging, (170.6510) Spectroscopy, tissue diagnostics, (170.3880) Medical and biological imaging

## Abstract

The statistical independence between the distributions of different chromophores in tissue has previously been used for linear unmixing with independent component analysis (ICA). In this study, we propose exploiting this statistical property in a nonlinear model-based inversion method. The aim is to reduce the sensitivity of the inversion scheme to errors in the modelling of the fluence, and hence provide more accurate quantification of the concentration of independent chromophores. A gradient-based optimisation algorithm is used to minimise the error functional, which includes a term representing the mutual information between the chromophores in addition to the standard least-squares data error. Both numerical simulations and an experimental phantom study are conducted to demonstrate that, in the presence of experimental errors in the fluence model, the proposed inversion method results in more accurate estimation of the concentrations of independent chromophores compared to the standard model-based inversion.

## 1. Introduction

Photoacoustic tomography is a non-invasive biomedical imaging technique [1] relying on the absorption of optical energy and the generation of ultrasound waves. It has a relatively low cost of implementation and has the advantage of combining the relatively large penetration depth and high resolution of ultrasound imaging with optical absorption based contrast which provides high specificity. In quantitative photoacoustic tomography, the unique optical absorption spectra of the chromophores are exploited in spectroscopic inversions of the multiwavelength photoacoustic images in order to estimate the concentration of each chromophore. The key endogenous chromophores of interest for quantitative photoacoustic tomography are oxy- and deoxyhaemoglobin, because the ratio of their concentrations is related to the blood oxygenation, which is an important physiological parameter. Spectroscopic techniques are also valuable tools for contrast-enhanced photoacoustic molecular imaging applications, where the detection and quantification of the local accumulation of genetically encoded probes and extrinsically administered contrast agents [2] can provide information on biological processes, drug delivery, disease development and treatment response.

Quantifying the chromophore concentrations is a challenging task because the absorbed optical energy density is not linearly related to the chromophore concentrations, but a product of the absorption coefficient and light fluence, which varies both spatially and spectrally and depends on the unknown chromophore concentrations. The model-based inversion scheme [3–8] is a quantification method that accounts for the effect of the fluence by including a numerical model of the fluence distribution in an iterative scheme to solve the inverse problem. Thus, it has the potential to provide accurate estimation of the absolute chromophore concentrations in complex tissue structures. However, in experimental settings, both the approximate nature of the model, and the difficulty in determining all the “known” model parameters, such as the absorption and scattering spectra and the intensity profile of the excitation beam, to high accuracy, lead to model-mismatch. This poses challenges on the practical implementation of model-based inversion schemes for *in vivo* imaging. Previous studies [5,8] have relied on reducing the number of unknown variables by segmenting the images into regions with piece-wise constant optical properties to increase the robustness of the inversion scheme. In this study, we exploit instead the statistical independence between certain chromophores to improve the accuracy and robustness of model-based inversion.

Statistical independence has been utilised to spectrally decompose the chromophores via independent component analysis (ICA) [9]. ICA is a fast and simple unmixing algorithm based on the assumption that the photoacoustic images are linear mixtures of the independent source components representing the chromophores. Glatz *et al* [10] showed that ICA can result in more accurate unmixing than a linear spectroscopic inversion. ICA has subsequently been used to unmix genetic reporters [11] and contrast agents targeted to apoptotic cells [12] or cells expressing selectin [13]. However, ICA is unable to estimate the absolute concentrations of the chromophores and it does not account for the nonlinear fluence distortion.

In this paper, we propose incorporating statistical independence as additional information in the nonlinear model-based inversion method. This involves including a measure of the independence in the error functional in addition to the least-squares data error. The aim is to reduce the quantification errors caused by inaccurate forward modelling of the fluence. The effect of using the statistical independence in the inversion is investigated using both numerical and experimental tissue mimicking phantoms and the quantification results are compared to the model-based inversion using only the data error.

## 2. Mutual information error functional

Detailed descriptions of statistical independence and its applications in imaging can be found in Refs [14,16,17]. However, as the concept has not yet been widely used in photoacoustic imaging, a brief summary of the essential aspects will be given here. The spatial distributions of certain biomarkers or molecular probes are statistically independent from other tissue chromophores in some cases of practical interest. Examples include fluorescent probes that can be targeted to disease-specific receptors whose spatial distribution is unrelated to that of the blood and the background tissue, and cells that have been genetically modified to express optical absorbers which can be found at locations independent from other absorbers. The statistical independence of two chromophores is related to the histogram of the values of the individual chromophore concentrations, and the joint histogram of the concentrations of both chromophores. The mathematical definition of statistical independence states that two random variables, **y**_1_ and **y**_2_, are statistically independent if their joint probability density function (PDF), *ρ*_**y**_1_,**y**_2__(*y*_1_, *y*_2_), is the product of their marginal PDFs, *ρ*_**y**_1__(*y*_1_) and *ρ*_**y**_2__(*y*_2_) [14], such that

(1) $$\rho_{y_{1},y_{2}}(y_{1},y_{2})=\rho_{y_{1}}(y_{1})\rho_{y_{2}}(y_{2}),$$

where *y*_1_ and *y*_2_ denote possible values of **y**_1_ and **y**_2_. The degree of independence between variables can be measured using the mutual information (MI). MI is an estimate of the amount of information one variable provides on another variable. Variables with higher independence have lower MI, which means that they contain less information about each other. In quantitative photoacoustic tomography, two chromophores are considered independent if the knowledge of the concentration of one chromophore at a location does not affect the estimate of the other chromophore’s concentration at the same location. For example, if a contrast agent is independent of the blood, then the estimation of the contrast agent concentration at a voxel does not in any way predict the blood concentration there. As a counter example, oxy- and deoxyhaemoglobin are not independent chromophores: if a voxel is found to contain a high concentration of deoxyhaemoglobin, then the likelihood that a high concentration of oxyhaemoglobin will be found at the same pixel increases, as the voxel is likely to represent a blood vessel. The MI, *I*, between **y**_1_ and **y**_2_ is given by

(2) $$I(y_{1},y_{2})=ℋ(y_{1})+ℋ(y_{2})-ℋ(y_{1},y_{2}),$$

where *ℋ*(**y***_k_*) and *ℋ*(**y**_1_, **y**_2_) are the entropy and the joint entropy of **y**_1_ and **y**_2_ respectively. They are defined by

(3) $$ℋ(y_{k})=-\int_{y_{k}}\rho_{y_{k}}(y_{k})\text{log}\rho_{y_{k}}(y_{k})\text{d}y_{k},$$

where *k* = 1 or 2, and

(4) $$ℋ(y_{1},y_{2})=-\int_{y_{1}}\int_{y_{2}}\rho_{y_{1},y_{2}}(y_{1},y_{2})\text{log}\rho_{y_{1},y_{2}}(y_{1},y_{2})\text{d}y_{1}\text{d}y_{2}.$$

Similarly, the MI between multiple random variables is defined by

(5) $$I(y_{1},\ldots ,y_{K})=\sum_{k=1}^{K}ℋ(y_{k})-ℋ(y_{1},\ldots ,y_{K}).$$

As indicated in Eqs. (2–4), the MI depends on the probabilities of the variables. We consider the concentrations of *K* independent chromophores **c**_1_, ..., **c***_K_* as continuous random variables with the PDFs *ρ*_**c**_1__, ..., *ρ*_**c**_*K*__. However, the underlying probabilities of the chromophore concentrations are unknown and therefore *ρ*_**c**_*k*__ needs to be estimated based on the instantiations of the random variable, which are the concentrations of the chromophore at different voxels [*c*_*k*,1_, ..., *c*_*k*,*M*_]. The total number of voxels, which is equal to the total number of instantiations, is denoted with *M*. Using these data points, the PDF can be approximated with a kernel density estimator. Conceptually, the kernel density estimation method involves placing a smoothly varying spread of values, or kernel, on the value of each data point. The probability is then approximated by the sum of all kernels, such that

(6) $${\overset{˘}{\rho }}_{c_{k}}(\xi_{k})=\frac{1}{M}\sum_{m=1}^{M}\kappa (\xi_{k}-c_{k,m})$$

and

(7) $${\overset{˘}{\rho }}_{c_{1},\ldots ,c_{K}}(\xi_{1},\ldots ,\xi_{K})=\frac{1}{M}\sum_{m=1}^{M}\prod_{k=1}^{K}\kappa (\xi_{k}-c_{k,m}),$$

where *ξ_k_* are the values at which the PDF is evaluated and *κ*(*x*) is a kernel function that satisfies *κ*(*x*) ≥ 0 and $\int_{-\infty }^{\infty }\kappa (x)\text{d}x=1$. The breve symbol (˘) is used to denote the estimation of a quantity. The motivation for using the kernel density estimator instead of a simple histogram approximation for the probabilities is that the former ensures continuity and differentiability of the estimated PDFs, provided that a continuous and differentiable kernel function is used. To satisfy those criteria, the Gaussian kernel is chosen for this study:

(8) $$\kappa (x)=\frac{1}{h\sqrt{2\pi }}\text{exp}\left(-x^{2}/2h^{2}\right),$$

where *h* is the kernel width, which determines the amount of smoothing in the estimations. It is calculated using the expression for the optimal kernel width normally distributed data, given by [15]

(9) $$h={\left(\frac{4}{3M}\right)}^{\frac{1}{5}}\sigma \approx 1.06\sigma M^{-\frac{1}{5}},$$

where *σ* is the standard deviation of the data.

To estimate the entropies of the chromophore concentrations, the PDF is evaluated at a set of equally spaced points denoted by [*ξ*_*k*,1_, ..., *ξ*_*k*,*Q*_], where *Q* is the total number of points. The integral in the definition of the entropy and the joint entropy for continuous variables in Eq. (3) and (4) can be approximated as a sum using the trapezium rule, such that

(10) $$\overset{˘}{ℋ}(c_{k})=-\sum_{q_{k}=1}^{Q}{\overset{˘}{\rho }}_{c_{k}}(\xi_{k,q_{k}})\;\text{log}\;{\overset{˘}{\rho }}_{c_{k}}(\xi_{k,q_{k}})\Delta \xi_{k}$$

and

(11) $$\overset{˘}{ℋ}(c_{1},\ldots ,c_{K})=-\sum_{q_{1},\ldots ,q_{K}=1}^{Q}{\overset{˘}{\rho }}_{c_{1},\ldots ,c_{K}}(\xi_{1,q_{1}},\ldots ,\xi_{K,q_{K}})\;\text{log}\;{\overset{˘}{\rho }}_{c_{1},\ldots ,c_{K}}(\xi_{1,q_{1}},\ldots ,\xi_{K,q_{K}})\prod_{k=1}^{K}\Delta \xi_{k}$$

where the summation symbol denotes a multiple sum of *q*_1_, ..., *q_K_* and Δ*ξ_k_* is the spacing between the sampling points for the PDF. Using the approximated entropies, the MI can be estimated by

(12) $$\overset{˘}{I}(c_{1},\ldots ,c_{K})=\sum_{k=1}^{K}\overset{˘}{ℋ}(c_{k})-\overset{˘}{ℋ}(c_{1},\ldots ,c_{K}).$$

To find the most independent chromophores requires minimising the MI between the chromophores, which can be done efficiently using a gradient-based optimisation approach. The partial derivative of the MI with respect to the chromophore concentration at each voxel is given by [18]

(13) $$\frac{\partial \overset{˘}{I}(c_{1},\ldots ,c_{K})}{\partial c_{k,m}}=\frac{\overset{˘}{ℋ}(c_{k})}{\partial c_{k,m}}-\frac{\partial \overset{˘}{ℋ}(c_{1},\ldots ,c_{K})}{\partial c_{k,m}}.$$

The first term in the right hand side of Eq. (13) is

(14) $$\frac{\partial \overset{˘}{ℋ}(c_{k})}{\partial c_{k,m}}=\frac{\partial \overset{˘}{ℋ}(c_{k})}{\partial {\overset{˘}{\rho }}_{c_{k}}}\frac{\partial {\overset{˘}{\rho }}_{c_{k}}}{\partial c_{k,m}},$$

where

(15) $$\frac{\partial \overset{˘}{ℋ}\left(c_{k}\right)}{\partial {\overset{˘}{\rho }}_{c_{k}}}=-\sum_{q_{k}=1}^{Q}\left[1+\text{log}\;{\overset{˘}{\rho }}_{c_{k}}(\xi_{k,q_{k}})\right]\Delta \xi_{k}$$

by the product rule, and

(16) $$\frac{\partial {\overset{˘}{\rho }}_{c_{k}}}{\partial c_{k,m}}=\kappa^{\prime }(\xi_{k,q_{k}}-c_{k,m}),$$

where *κ′* denotes the derivative of *κ*, given by

(17) $$\kappa^{\prime }(\xi_{k,q_{k}}-c_{k,m})=\frac{\xi_{k,q_{k}}-c_{k,m}}{h^{2}}\kappa (\xi_{k,q_{k}}-c_{k,m})$$

for the Gaussian kernel. Similarly, the second term in Eq. (13) is

(18) $$\frac{\partial \overset{˘}{ℋ}(c_{1},\ldots ,c_{K})}{\partial c_{k,m}}=\frac{\partial \overset{˘}{ℋ}(c_{1},\ldots ,c_{K})}{\partial {\overset{˘}{\rho }}_{c_{1},\ldots ,c_{K}}}\frac{\partial {\overset{˘}{\rho }}_{c_{1},\ldots ,c_{K}}}{\partial c_{k,m}},$$

where

(19) $$\frac{\partial \overset{˘}{ℋ}(c_{1},\ldots ,c_{K})}{\partial {\overset{˘}{\rho }}_{c_{1},\ldots ,c_{K}}}=-\sum_{q_{1},\ldots q_{K}=1}^{Q}\left[1+\text{log}{\overset{˘}{\rho }}_{c_{1},\ldots ,c_{K}}\left(\xi_{1,q_{1}},\ldots ,\xi_{K,q_{K}}\right)\right]\prod_{i=1}^{K}\Delta \xi_{i}$$

and

(20) $$\frac{\partial {\overset{˘}{\rho }}_{c_{1},\ldots ,c_{K}}}{\partial c_{k,m}}=\kappa \prime (\xi_{k}-c_{k,m})\prod_{i=1,i\neq k}^{K}\kappa (\xi_{i}-c_{i,m}).$$

## 3. Model-based inversion with statistical independence

The first step in the model-based inversion scheme used in this study is to make an initial guess for the unknown chromophore concentrations. This initial guess and the known specific absorption spectra *α* are used to calculate the absorption coefficient, $\mu_{a}=\sum_{k}\alpha_{k}c_{k}$. The fluence, Φ, is then modelled using the diffusion approximation to the radiative transfer equation for the distribution of the absorption coefficient and the scattering coefficient. The scattering coefficient is assumed to be known in this study. Using the modelled fluence, the absorption coefficient and the Grüneisen parameter Γ, the modelled initial pressure $p_{m,\lambda_{n}}^{\mathrm{model}}$ at voxel *m* and wavelength *λ_n_* is calculated based on

(21) $$p_{m,\lambda_{n}}^{\mathrm{model}}=S\Gamma_{m}\Phi_{m,\lambda_{n}}\mu_{a\_m,\lambda_{n}},$$

where *S* is the system calibration factor that depends on the acoustic response of the sensors, which can be assumed to be spatially homogeneous and independent of the optical wavelength. The data error, *ε_d_*, is defined as the sum of squared differences between the modelled and the measured initial pressure, $p_{m,\lambda_{n}}^{\mathrm{meas}}$, and hence the minimisation problem is given by

(22) $$\underset{c_{1},\ldots ,c_{K_{t}}}{\text{argmin}}\;\varepsilon_{d}(c_{1},\ldots ,c_{K_{t}})=\sum_{n=1}^{N}\sum_{m=1}^{M}{\left[p_{m,\lambda_{n}}^{\mathrm{model}}(c_{1},\ldots ,c_{K_{t}})-p_{m,\lambda_{n}}^{\mathrm{meas}}\right]}^{2},$$

where the total number of chromophores, wavelengths and voxels are denoted by *K_t_*, *N*, and *M* respectively, and **c***_i_* denotes the *i*^th^ chromophore. By iteratively updating the values of the chromophore concentrations until *ε_d_* is minimised, accurate quantification can be achieved in an idealised scenario. However, in practical applications, model-mismatch arises due to the approximations in the model and uncertainty in the model parameters (which are typically determined experimentally). This leads to the minimum of the data error occurring at erroneous chromophore concentrations, and results in inaccurate quantification. Unlike the data error, the statistical independence is a property of the distribution of the chromophores alone, rather than a function of the forward modelling. Therefore, the errors in the fluence model do not affect the MI between the chromophore concentrations, which will always have a minimum at the true solution, provided that the chromophores are statistically independent. Hence, by including a term representing the MI between the independent chromophores in the error functional, the quantification errors can be reduced. The new minimisation problem with both the data error and the MI is given by

(23) $$\underset{c_{1},\ldots ,c_{K_{t}}}{\text{argmin}}\;\varepsilon_{d+MI}(c_{1},\ldots ,c_{K_{t}})=\sum_{n=1}^{N}\sum_{m=1}^{M}{\left[p_{m,\lambda_{n}}^{\mathrm{model}}(c_{1},\ldots ,c_{K_{t}})-p_{m,\lambda_{n}}^{\mathrm{meas}}\right]}^{2}+\gamma \overset{˘}{I}(c_{1},\ldots ,c_{K}),$$

where *γ* is the weight parameter for the MI, and total number of chromophores may be larger than or equal to the number of independent chromophores, *K_t_* ≥ *K*.

## 4. Generating multiwavelength photoacoustic images of tissue mimicking phantoms

The accuracy of the quantification using *ε*_*d*+*MI*_ compared to *ε_d_* was investigated using experimental and numerical tissue mimicking phantoms containing aqueous solutions of copper(II) chloride dihydrate, nickel(II) chloride hexahydrate and black India ink (Winsor & Newton, London, UK), which represent different absorbers in the tissue, and Intralipid, which provides scattering in the medium. The copper(II) chloride dihydrate and nickel(II) chloride hexahydrate will be referred to as CuCl_2_ and NiCl_2_. For both the numerical and the experimental phantom, the distributions of CuCl_2_ and NiCl_2_ are arranged such that they are statistically independent of each other.

### 4.1. Experimentally acquired images

**Fig. 1:**
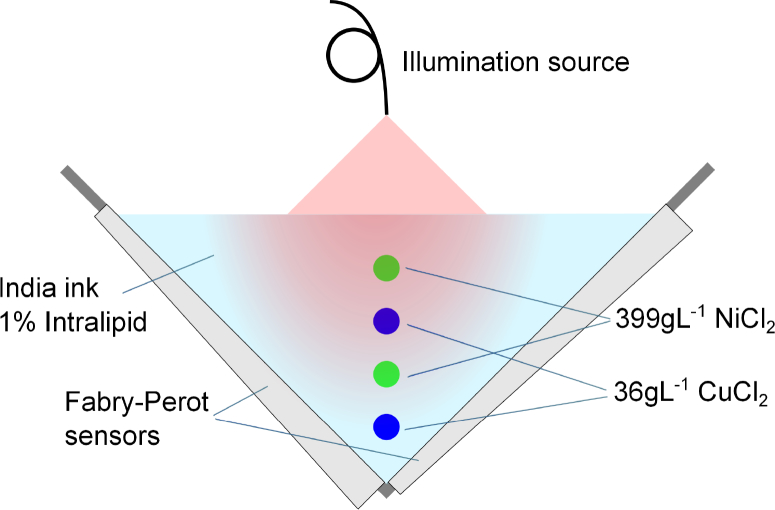
Experimental set-up and phantom structure. The four tubes containing CuCl_2_ or NiCl_2_ are fixed in a vertical column and submerged in the India ink and Intralipid solution. Two orthogonal Fabry-Perot interferometer sensors are used for increased detection aperture. The fibre tip at the top of the phantom delivers the pulsed excitation beam.

A schematic of the experimental set-up is shown in Fig. 1

**Fig. 2:**
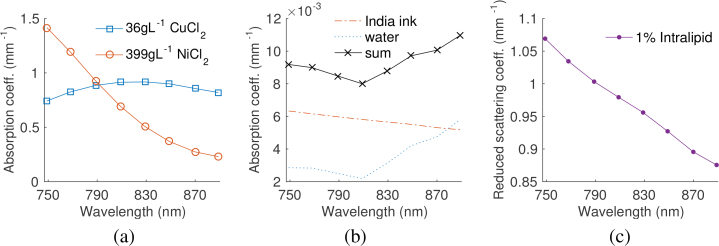
(a) The absorption coefficients of 36gL^−1^ CuCl_2_ (squares) and 399gL^−1^ NiCl_2_ (circles). (b) The absorption coefficient of the background solution (crosses), which is a sum of the absorption of water [20] (dotted) and the India ink (dashed). (c) The scattering amplitude of 1% Intralipid as a function of wavelength [21]. A spectrophotometer (Lambda 750S, Perkin Elmer) was used to measure the transmittance of CuCl_2_, NiCl_2_ and India ink in order to determine their absorption spectra. Reprinted from [22].

. The tissue mimicking phantom consists of four polythene tubes with 0.58mm inner diameter and 0.19mm wall thickness (Scientific Laboratory Supplies Ltd, Nottingham, UK) submerged in a background solution of highly diluted India ink and 1% (w/v) Intralipid, which give rise to an absorption and scattering amplitude comparable to that of typical biological tissue [19]. The tubes are arranged in a line at depths of approximately 3.6, 6.1, 8.1 and 9.8mm from the top surface of the phantom, which are all within the diffusive regime. The first and third tube from the top contain 399gL^−1^ NiCl_2_ and the second and the fourth tube contain 36gL^−1^ CuCl_2_. The absorption spectra of the chromophores and scattering spectrum of Intralipid are shown in Fig. 2

. The CuCl_2_ and NiCl_2_ are statistically independent of each other in this phantom, which is clear from the fact that they are contained in distinct regions that are spatially separated. (However, the spatial separation is not a necessary criterion for statistical independence. For example, CuCl_2_ and NiCl_2_ are both also statistically independent from water, even though they can be found at the same locations.)

The phantom is imaged in a V-shaped photoacoustic imaging system [24,25] consisting of two orthogonal Fabry-Perot interferometer sensors [26]. This sensor geometry increases the detection aperture of the photoacoustic signals compared to a single planar detector array and hence reduces the limited-view artefacts [25]. The fibre tip was positioned vertically above the phantom to deliver the pulsed excitation light from a Nd:YAG-pumped optical parametric oscillator (GWU, Spectra-Physics, Santa Clara, USA) with 10Hz repetition rate and a pulse energy of 15-19mJ depending on wavelength. The phantom was imaged at 8 wavelengths with equal spacing between 750nm and 890nm by recording the photoacoustic time series at a 13×13mm^2^ area with 100*μ*m step size for both sensors. A small fraction of the light was directed to an integrating sphere to measure the pulse energy, which was used to normalise the measured signals. The beam position and the spatial distribution of the beam intensity at the surface of the phantom was found by acquiring an additional photoacoustic image at 780nm of a transparent sheet with a printed grid of highly absorbing dots (with 1mm grid spacing) which was resting on the surface. Based on the reconstruction of this image, the illumination source was approximated as a Gaussian beam with a 1/e diameter of 6.6mm.

**Fig. 3:**
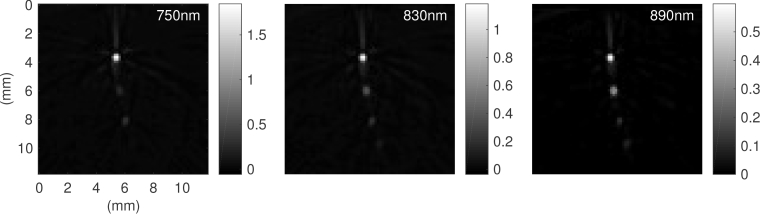
The 2D cross-sectional slices of the 3D reconstructed photoacoustic images which are used for the optical inversion at wavelengths 750, 830 and 890nm. The size of this region of interest is 12×12mm^2^ and the element spacing is 166*μ*m. As expected, the intensity of the tubes decreases with depth for all wavelengths due to the decay of the fluence.

The iterative time reversal method [27, 28] was used to reconstruct 3D images from the photoacoustic time series. A 2D cross-sectional 12×12mm^2^ region of interest centred at the tubes was used for the optical inversion, and the dimension was reduced to 72×72 pixels to reduce the computational time and memory requirements. The 2D slices are shown for three wavelengths in Fig. 3

.

### 4.2. Numerically simulated images

**Fig. 4:**
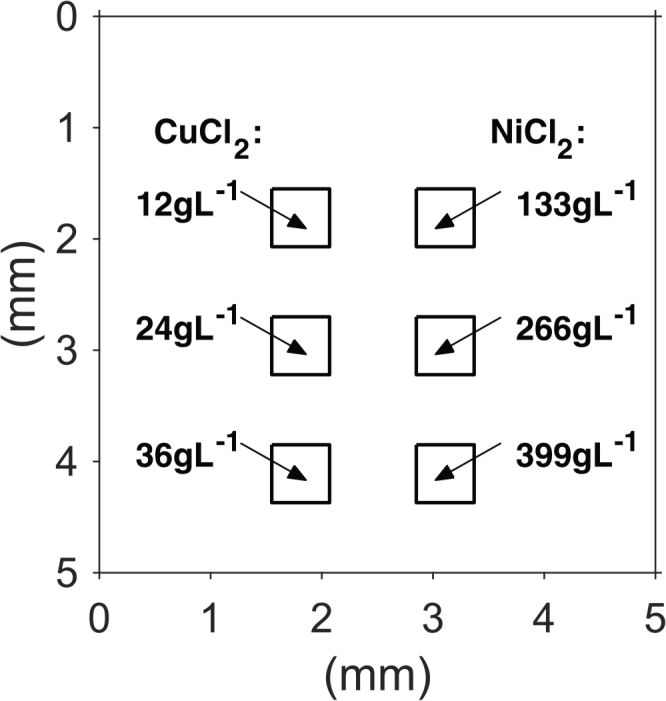
Diagram of the 2D numerical phantom. The phantom contains regions with different concentrations of CuCl_2_ and NiCl_2_. The background region contains India ink and Intralipid.

The numerical phantom has an element spacing of 100*μ*m and represents a 5×5mm^2^ area with six insertions arranged in two columns, as illustrated in Fig. 4

. The left column of insertions represents solutions of CuCl_2_ with concentrations 12, 24 and 36gL^−1^ in increasing order, where the top insertion has the lowest concentration. The right column of insertions contain solutions of NiCl_2_ with concentrations 133, 266 and 399gL^−1^, also in increasing order from the top insertion. The phantom is designed with increasing concentrations at the insertions with depth to improve the signal to noise ratio at the deeper insertions. The absorption of CuCl_2_ and NiCl_2_ are based on the measured spectra shown in Fig. 2(a) and assumed to follow linear dependence on concentration. The concentrations are chosen such that the average absorption of both columns is 0.52mm^−1^ over the wavelength range between 750nm to 890nm, which is similar to the absorption of blood over the same range of wavelengths. Water is present in the whole phantom and the background region outside the insertions represents a solution of India ink and Intralipid, which gives rise to the same absorption and scattering amplitude as shown in Fig. 2(b–c).

The top surface of the domain was illuminated with a radially-symmetric light source with a Gaussian intensity profile with a 1/e width of 3mm. The light fluence distributions for the same 8 wavelengths as the experimental measurement in Sec. 4.1 were simulated using the diffusion approximation with the MATLAB software Toast++ [23]. The system calibration factor and the Grüneisen parameter are assumed to be known and equal to one, and the acoustic reconstruction is assumed to be perfect, such that the simulated photoacoustic images were equal to the product of the fluence and the absorption coefficient. Gaussian noise with an amplitude equal to 10% of the mean of the data, which was comparable to the magnitude of the noise in the experimental images (Sec. 4.1), was added to the simulated images.

## 5. Inverting for the chromophore concentrations

To investigate the effect of incorporating the MI term, the model-based inversion scheme was applied to the simulated and the experimentally acquired multiwavelength 2D photoacoustic images using both *ε_d_* and *ε*_*d*+*MI*_ error functionals. The error functionals were minimised with the limited-memory BFGS [29] algorithm which searches for the optimal chromophore concentrations using the functional gradients of the data error term [3] and the MI term [18]. The unknown parameters in the inversion are the concentrations of CuCl_2_, NiCl_2_, India ink and water, and the known model parameters are the absorption spectra, the scattering distribution, the system calibration factor, the Grüneisen parameter and the light source position and width. The unknown chromophore concentrations were initialised with a spatially homogeneous value equal to the true concentration at the background. The iterative update was run for 300 iterations for the inversion of both the simulated and the experimental images using *ε_d_* or *ε*_*d*+*MI*_. For all inversions using *ε*_*d*+*MI*_, the MI was calculated between CuCl_2_ and NiCl_2_ and the weight parameter *γ* of the MI term was set to zero for the first 200 iterations to avoid the algorithm being trapped in the local minima of the MI term. The difference in computation time for *ε*_*d*+*MI*_ and *ε_d_* was negligible. The computationally efficient calculation of the MI term and its gradient was achieved using fast Fourier transforms [30], which takes advantage of the fact that Eqs. (6) and (7) have convolution structures.

### 5.1. Effect of model-mismatch

**Fig. 5:**
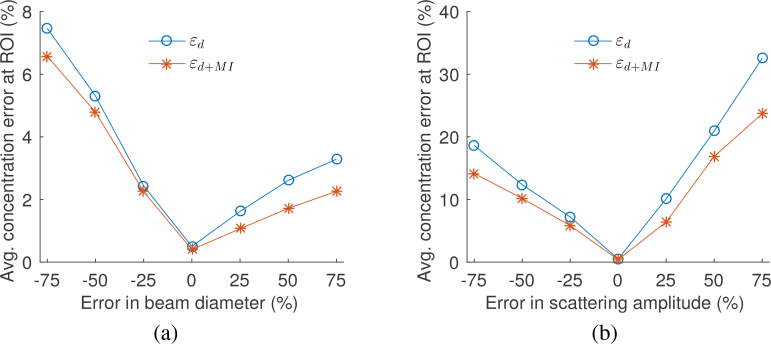
The average errors of the estimated concentrations of CuCl_2_ and NiCl_2_ at the insertions as a function of errors in (a) the beam diameter or (b) the scattering amplitude in the inversion. As expected, the quantification errors increase for larger errors in the beam diameter or scattering amplitude. However, the inversions using *ε*_*d*+*MI*_ (asterisks) result in smaller errors compared to using *ε_d_* (circles) for all data points. The individual errors for CuCl_2_ and NiCl_2_ show similar general trends as the average of the two. The average errors outside the ROI are <2% for inversions using both *ε_d_* and *ε*_*d*+*MI*_. Reprinted from [22].

Two case studies were conducted using the simulated images to investigate the effect of the uncertainty in different model parameters on the quantification accuracy. In the first case, the beam diameter was set to be up to 75% smaller or larger than the true value in the inversion. In the second case, an error of up to ±75% was included in the scattering amplitude. The average errors of the estimated concentrations of CuCl_2_ and NiCl_2_ at the insertions (ROI) using the erroneous beam diameter are shown in Fig. 5(a)

, where the circles and asterisks correspond to the inversions using *ε_d_* or *ε*_*d*+*MI*_ respectively. The results show that the increase in the percentage error in the beam diameter leads to larger quantification errors, as expected. The quantification errors for the simulated images are relatively small because only one model parameter contains error at the time. In an experimental setting, there is likely to be a combination of modelling errors, resulting in larger quantification errors. Including the MI term results in a reduction in error compared to using only the standard data error for all data points.

The inaccuracies in the scattering amplitude used in the inversion resulted in similar trends for the quantification error, as shown in Fig. 5(b). The errors are generally larger in Fig. 5(b) than 5(a), which suggests that the changes in scattering amplitude have a larger impact on the fluence distribution than changes in the beam diameter for this numerical phantom. Nonetheless, using *ε*_*d*+*MI*_ is shown to provide more accurate estimations compared to using *ε_d_* also for this case. The relative improvement in accuracy varied between 37% and 8% with an average of 22% over all data points for both cases.

### 5.2. Experimental results

The multiwavelength experimental images were divided by the calibration factor and the spatially varying Grüneisen parameter before the inversion. The calibration factor was determined using a forward simulation with the true concentrations. The Grüneisen parameter of aqueous solutions of CuCl_2_ and NiCl_2_ are both known to increase with concentration and are given by

(24) $$\Gamma_{i}=\Gamma_{H_{2}O}(1+\beta_{i}c_{i}),\;i={\text{CuCl}}_{2}\;\text{or}\;{\text{NiCl}}_{2}$$

where Γ_*H*_2_*O*_ is the Grüneisen parameter for water, and *β_i_* are 5.80×10^−3^Lg^−1^ and 2.25×10^−3^Lg^−1^ for CuCl_2_ or NiCl_2_ respectively [31]. In practical applications, where the true concentrations are unknown, the calibration factor can be obtained by measuring the acoustic sensitivity of the sensors [32], and the Grüneisen parameter can be included in the model as a parameter that is linearly dependent on the estimated chromophore concentrations [5,32].

**Fig. 6:**
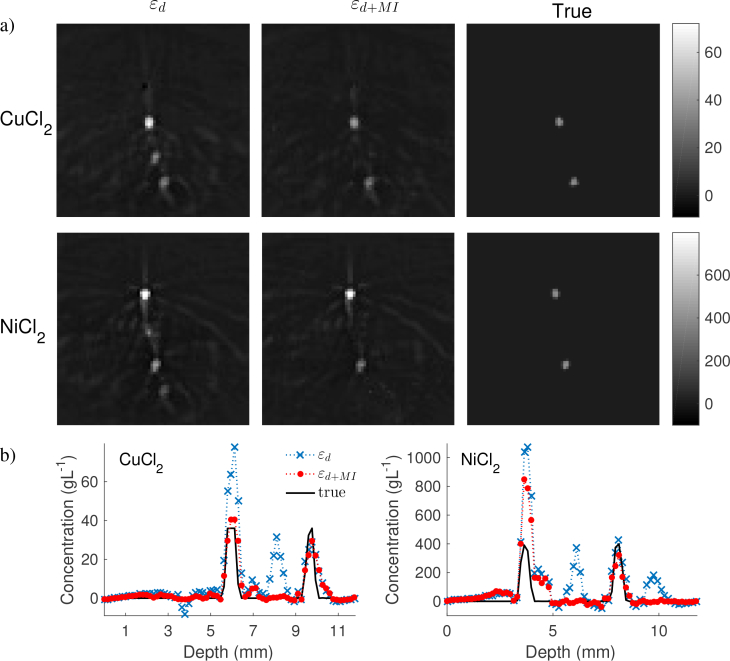
(a) The estimated concentrations of CuCl_2_ (top row) and NiCl_2_ (bottom row) in units of gL^−1^. The results from the inversions using *ε_d_* (left column) show overestimation of the the upper tubes and large cross-talk errors, while using *ε*_*d*+*MI*_ results in more accurate quantification without cross-talk errors. The true concentrations are shown in the column to the right for comparison. (b) The estimated concentration of the CuCl_2_ (top) and NiCl_2_ (bottom) using *ε_d_* (crosses) and *ε*_*d*+*MI*_ (circles) along a line across the tubes. The solid curves represent the true concentrations.

The results from the model-based inversion of the experimental data are presented in Fig. 6

. The estimated concentrations of CuCl_2_ (top row) and NiCl_2_ (bottom row) using *ε_d_* and *ε*_*d*+*MI*_ are shown in the left and centre columns respectively in Fig. 6(a), while the true concentrations are shown in the right column. The colour scale indicates the concentrations in units of gL^−1^. Figure 6(b) compares the estimated with the true concentrations along a line profile across the tubes. The average estimated and expected concentrations for each tube are presented in Table 1

**Table 1:** The average estimated and true concentrations of CuCl_2_ (left) and NiCl_2_ (right) in gL^−1^ for each tube. The largest improvements using *ε*_*d*+*MI*_ are mostly seen for the tubes that are not expected to contain the relevant chromophore, as they suffer from significant cross-talk errors when *ε_d_* is used. The average expected concentrations are lower than the true concentrations in the solutions due to the interpolation from the original images.

CuCl_2_	*ε_d_*	*ε*_*d*+*MI*_	Expected
Tube 1	−3	−1	0
Tube 2	60	35	31
Tube 3	26	0	0
Tube 4	27	25	31

NiCl_2_	*ε_d_*	*ε*_*d*+*MI*_	Expected
Tube 1	860	698	345
Tube 2	185	−5	0
Tube 3	367	258	352
Tube 4	167	14	0

. The inversions using *ε_d_* resulted in high overestimation of CuCl_2_ in the second tube and NiCl_2_ in the first tube, where the estimated concentrations are 94% and 149% larger than the true values respectively. There are also large cross-talk errors in the estimation of both contrast agents. This is most clearly seen for the estimated CuCl_2_ concentration, where the third tube shows a high false-positive concentration with comparable magnitude to the concentration in the fourth tube. The accuracy of the quantification is significantly improved when the MI term is included in the error functional. The cross-talk errors for both CuCl_2_ and NiCl_2_ are almost completely removed when *ε*_*d*+*MI*_ is used. The absolute concentrations of the CuCl_2_ is estimated accurately with an error of 3gL^−1^ on average for the four tubes. The overestimation of the NiCl_2_ concentration in the top tube remains present with *ε*_*d*+*MI*_. However, this overestimation error is reduced when *ε*_*d*+*MI*_ is used compared to *ε_d_*.

## 6. Discussion

Minimising the MI as well as the data error led to improved quantification accuracy for both simulated and experimental multiwavelength photoacoustic images, compared to using the data error alone. In the numerical study, despite the significant fractional decrease of the quantification errors using *ε*_*d*+*MI*_, in absolute terms, the error was decreased only by a few per cent. This was mainly due to the fact that only one model parameter was erroneous in each inversion, while all other assumptions in the model were accurate, which resulted in relatively small quantification errors, even when only *ε_d_* was used. In the experimental study, on the other hand, a combination of different types of modelling errors was likely to have been present simultaneously, leading to poorer quantification results in the absence of the MI term. The main causes of model-mismatch may be due to the limited size of the modelled domain, which does not account for the backscattered light from outside of the domain, and the 2D modelling of the light fluence, which assumes that the light source is constant in the direction along the tubes, while in the experiment, the beam was of circular cross-section. Other possible errors may include uncertainty in the scattering spectra and amplitude, as different values have been reported in the literature [21,33]. These errors in the model affect the calculation of $p_{m,\lambda_{n}}^{\mathrm{model}}$, which consequently also affect *ε_d_*. The MI is not affected by the fluence modelling errors, because MI is calculated based on only the distribution of the estimated chromophore concentrations in each iteration, and does not require the forward modelling of $p_{m,\lambda_{n}}^{\mathrm{model}}$. Therefore, the quantification errors were greatly reduced when *ε*_*d*+*MI*_ was used in the experimental study. These results suggest that incorporating the statistical independence can improve the robustness of model-based inversion schemes for independent chromophores and thus potentially enhance their applicability to pre-clinical or clinical imaging studies.

In order to obtain accurate results with the inversion using *ε*_*d*+*MI*_, it is necessary to use an appropriate weight parameter *γ* for the MI term. The weight was determined through manual trial and error. The solution was continuously dependent on the weight parameter, in the sense that small changes in the weight parameter resulted in small changes in the solution. The same weight was used for all inversions of the simulated and the experimental data, despite the differences in the data and/or the model parameters. This suggests that the concentration estimates were not highly sensitive to small variations of the weighting of the MI term around this value and, although non-trivial [34, 35], it may be possible to develop a general method for finding the optimal weight parameter for different types of applications.

The 2D fluence model based on the diffuse approximation assumes that the features are constant in the third dimension and located at depths within the diffusive regime. These assumptions are appropriate for the phantom geometry used in this study. However, full 3D fluence modelling will be required for applications of the model-based inversion in biological tissue with complex structures. More accurate modelling of the fluence for the superficial layer can be achieved by incorporating the delta-Eddington approximation [5] or using the radiative transfer equation [36]. The calculation of the MI can be straightforwardly extended to 3D without causing significant increase in computation time. Furthermore, using the MI term in the error functional is also compatible with other regularisation methods such as the total-variation regulariser [37–39].

## 7. Conclusion

We proposed exploiting the statistical independence between certain chromophores in the model-based inversion method by minimising the MI between the independent chromophores in addition to the data error. The improvement in the accuracy of the estimated chromophore concentrations was demonstrated using both numerical simulations and an experimental phantom. The results suggest that the sensitivity of the model-based inversion to model-mismatch can be reduced by incorporating the additional information of statistical independence. Thus, the robustness and hence usefulness of the inversion scheme can potentially be improved for *in vivo* imaging experiments.
